# Nitrogen fertilizer application rates and ratios promote the biochemical and physiological attributes of winter wheat

**DOI:** 10.3389/fpls.2022.1011515

**Published:** 2022-11-15

**Authors:** Muhammad Saleem Kubar, Chao Wang, Rana Shahzad Noor, Meichen Feng, Wude Yang, Kashif Ali Kubar, Khalilullah Soomro, Chen Yang, Hui Sun, Mohamed E. Hasan, Walid F. A. Mosa

**Affiliations:** ^1^ College of Agriculture, Shanxi Agricultural University, Taigu Jinzhong, China; ^2^ Department of Agriculture, Biological, Environment and Energy Engineering, College of Engineering, Northeast Agricultural University, Harbin, China; ^3^ Faculty of Agricultural Engineering and Technology, PMAS-Arid Agriculture University, Rawalpindi, Pakistan; ^4^ Faculty of Agriculture, Lasbela University of Agriculture, Water and Marine Sciences, Uthal, Pakistan; ^5^ Department of Plant Pathology, Sichuan Agricultural University, Chengdu, China; ^6^ Bioinformatics Department, Genetic Engineering and Biotechnology Research Institute, University of Sadat City, Sadat City, Egypt; ^7^ Plant Production Department (Horticulture- Pomology), Faculty of Agriculture, Saba Basha, Alexandria University, Alexandria, Egypt

**Keywords:** winter wheat, nitrogen fertilizer, photosynthetic traits, chlorophyll content, (SPAD)

## Abstract

Improper optimization of the rates and ratios of nitrogen application reduces grain yields and increases the nitrogen loss, thereby affecting environmental quality. In addition, scarcer evidence exists on the integrative approach of nitrogen, which could have effects on the biochemical and physiological characteristics of wheat. Treatments were arranged as nitrogen (N) rates of 00, 75, 150, 225, and 300 kg ha^−1^ in the main plots, and different nitrogen ratios were organized in subplots at 5:5:0:0 and 6:4:0:0, which were applied at the sowing, jointing, flowering, and grain filling stages. The results revealed that 225 kg N ha^−1^ significantly enhanced the stomatal conductance (*G*_s_), photosynthetic rate (*P*_n_), intercellular CO_2_ (*C*_i_), transpiration rate (*T*_r_), and total chlorophyll by 28.5%, 42.3%, 10.0%, 15.2%, and 50%, receptively, at the jointing stage in comparison to the control (0 kg N ha^−1^). Nitrogen application of 225 kg ha^−1^ increased the soil–plant analysis development (SPAD) value and the chlorophyll a, chlorophyll b, and carotenoid contents of winter wheat under the 6:4:0:0 ratio. The trend of the photosynthetic characteristics was observed to be greater at the 6:4:0:0 fertilization ratio compared to that at 5:5:0:0. The photosynthetic rate was significantly associated with the biochemical and physiological characteristics of winter wheat. In conclusion, the nitrogen dose of 225 kg ha^−1^ and the ratio of 6:4:0:0 (quantity applied at the sowing, jointing, flowering, and grain filling stages) effectively promoted the photosynthetic and other physiological characteristics of winter wheat.

## Introduction

Nitrogen management is considered one of the most vital factors affecting wheat growth (Chowdhury et al., 2021), phenology, and grain yield (Sabagh, 2021). Nitrogen fertilizer, when supplied at appropriate rates, plays a significant role in enhancing crop productivity (Chandio et al., 2022). Good nitrogen fertilization practices, including recommended methods and rates, are extremely vital not only for increasing the crop productivity but also for preserving the soil and eco-friendly health (Panhwar et al., 2019; Shah et al., 2019). However, insufficient application of synthetic fertilizers, mainly nitrogenous fertilizers, has a negative impact on the growth and yield of crops; it is expected that cumulative N fertilization may cause an enhancement of 23%–60% of N_2_O emissions by 2030 (FAO and WHO, 2009). Combining the physiological, chemical, and morphological traits responsible for inherent or environment-induced differences in the plant growth or yield requires thorough growth analysis (Hawkesford et al., 2013; Yingkui et al., 2016). The hypothetical higher limit for the active productivity of plant photosynthesis has been expected from a comprehensive stepwise investigation of the biophysical and biochemical substitute practices to be approximately 4.6% for C3 and 6.0% for C4 plants (Shi and Yu, 2008). These assessments adopted a leaf temperature of 30°C, as well as an atmospheric CO_2_ of 387 ppm, and were calculated comparative to the occupied planetary spectrum at the Earth’s surface. These productivities would be marginally more than twofold when calculated relative to simply the photosynthetically active emission (i.e., 400–700 nm) (Jialing and Wu, 2018). A plant’s pigment system is a foundation for the photosynthetic alteration of solar energy to biochemical bond energy. The major photosynthetic pigments are chlorophylls, while carotenoids (Car) hand over extra energy to chlorophylls (light-collecting function) and take off surplus energy from them (photoprotective function) (Hawkesford et al., 2013; Zuliang et al., 2018). Photosynthesis is the critical source of biomass in plants (Jialing and Wu, 2018). The biomass of plants is also related to the leaf area index because of its impact on light capture (Man et al., 2015; Mingnanet et al., 2017).

One of the acknowledged global changes as a result of rising CO_2_ is how it interacts with a plant’s features, such as a decrease in the transpiration rate or stomatal conductance and an increase in light usage efficiency (Li., 2018). According to data provided, CO_2_ caused a 31% increase in the saturated light needed for leaf photosynthesis and a 28% regular adjustment to photosynthetic carbon. However, full stress conditions such low and nitrogen depletion caused the stomatal conductance to fall and the net photosynthetic rate to decrease (Zuliang et al., 2018). For plant species such as maize, sorghum, sugarcane, and cereal grains including wheat, rice, and barley, a similar trend of 20% lower stomatal conductance was evident. In wheat, nitrogen fertilization boosted the net photosynthetic rate and improved the photosynthetic pigment characteristics. Previous research has shown a correlation between the leaf nitrogen content and the photosynthetic capacity (Li et al., 2018; Fan et al., 2011).

Studies have been conducted on the growth and biomass characteristics of winter wheat (Svetla et al., 2016; Xu et al., 2018; Hirel et al., 2011); however, studies on the integrative approaches of the effects of the rates and ratios on their biochemical and physiological traits are scarce (Yang et al., 2017; Oldfield et al., 2019; Skudra and Raza 2017). It is also interesting that the leaf chlorophyll content in plant leaves provides an enhanced approximation of the potential yield compared to the nitrogen concentration of leaves (Xing et al., 2018; Sahar et al., 2012). A few pieces of evidence exist regarding the effect of nitrogen management on photosynthetic characteristics, total dry biomass, and grain yield, as well as the relationship between grain yield and photosynthetic characteristics. Additionally, the economic impact of nitrogen management on the biomass production, chlorophyll content, photosynthetic rate, and nitrogen uptake efficiency of winter wheat crop has rarely been examined. Hence, this study was conducted to explore the effects of the nitrogen percentages and ratios on the biochemical and physiological characteristics of winter wheat.

## Materials and methods

### Experimental locations

**Figure 1:**
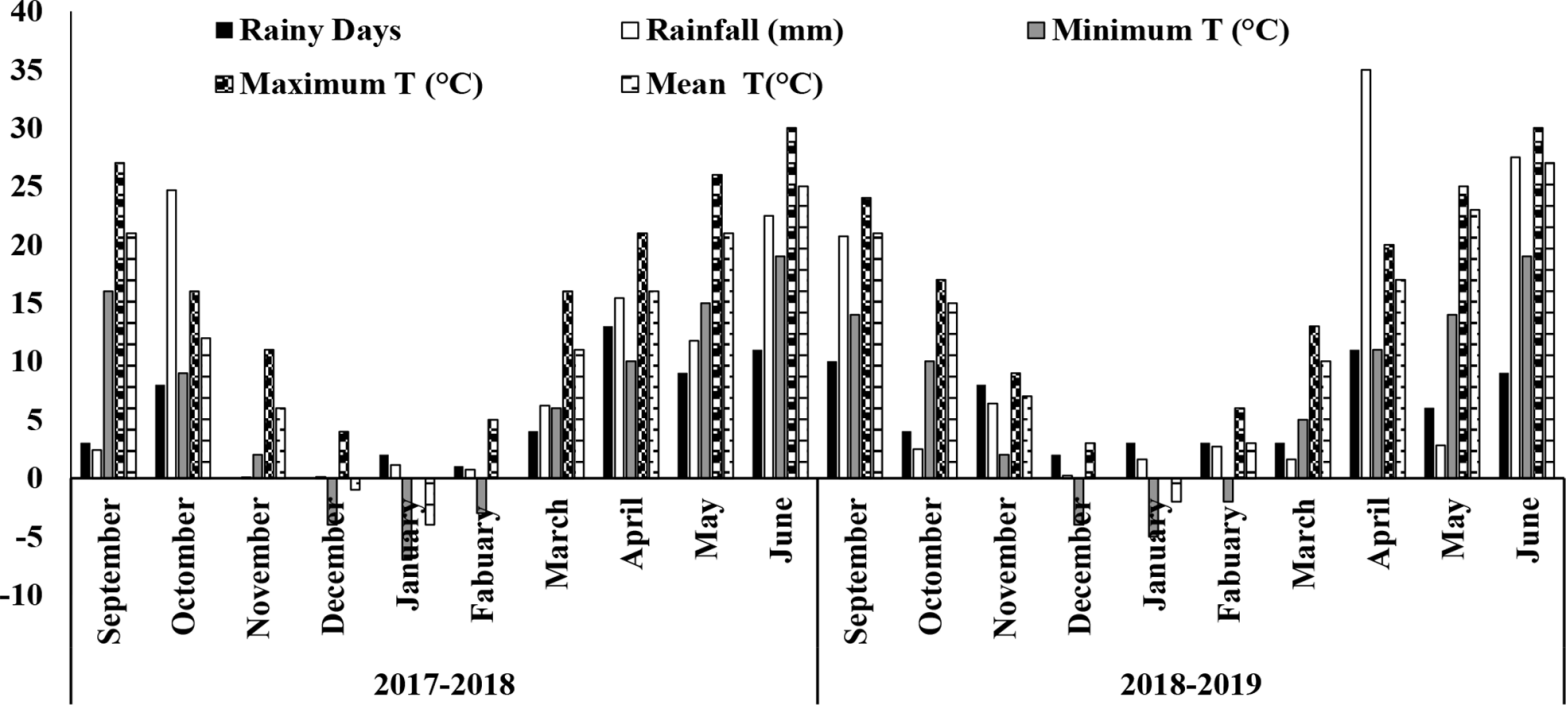
Monthly rainy days, rainfall, minimum temperature, maximum temperature, and mean temperature in 2017–2018 and 2018–2019 at Shanxi Agricultural University.

Field trials were conducted during two consecutive sowing seasons, 2017–2018 and 2018–2019, at the Taigu experimental farming station of Shanxi Agricultural University, Shanxi Province, China (N 37°25′, E 112°33′). The research area has a temperate continental monsoon climate with a mean yearly temperature of 12°C–13°C and a mean yearly rainfall of 442–600 mm. The potential evapotranspiration is from 1840.2 to 1872.2 mm, while the sunlight period is determined as 2,672–2,697 h. The research area is a mountainous arid field with a semiarid climate in the Northeast Loess Plateau, where 60%–70% of rainfall followed in the seasonal months through the fallow season (from July to August). The soil texture at the research site is clay loam. Details of the soil nutrients status is presented in **Table 1**. Weather data, including the minimum, maximum, and mean temperature, and the monthly average rainfall and rainy days are presented in **Figure 1**.

**Table 1:** Soil properties prior to the experiments at the farming station of Shanxi Agricultural University.

Year	Total N (g kg^−1^)	Total P (g kg^−1^)	Total K (g kg^−1^)	SOM (g kg^−1^)	pH
2017–2018	51.12	19.34	143.26	7.98	7.7
2018–2019	49.09	16.21	134.06	7.56	7.2

SOM, soil organic matter.

### Treatment detail

The trials were arranged in a split-plot design with three replications. The main plots included a control (no fertilizerapplied) means 0 (zero) and plots with total nitrogen (N) levels of 75, 150, 225, and 300 kg ha^−1^. Subplots received the following nitrogen ratios: 5:5 (50%, 50%) and 6:4 (60%, 40%). The labeling was as follows: 50%:50%:0% 0% (labeled as 5:5:0:0), 50%:0%:50%:0% (labeled as 5:0:5:0), and 50%:0%:0%:50% (labeled as 5:0:0:5) and 60%:40%:0%:% (labeled as 6:4:0:0), 60%:0%:40%:0% (labeled as 6:0:4:0), and 60%:0%:0%:40% (labeled as 6:0:0:4). The nitrogen fertilizer was applied as bottom and topdressing during the different growing stages of winter wheat in two ways: one was at 50% sowing time + 50% at jointing, 50% sowing time + 50% at the flowering stage, and 50% sowing time + 50% at the grain filling stage, while the other was at 60% at sowing time + 40% at the jointing stage, 60% at sowing time + 40% at the flowering stage, and 60% at sowing time + 40% at the grain filling stage. There were 75 plots, with the size of each experimental plot being 30 m^2^ (6 m × 5 m). Each treatment had three replications. Urea (46.4%) was used as the nitrogen source for the experimental field and was applied before sowing of the crop. Phosphorus was applied as triple super phosphate (16%) at 120 kg ha^−1^ and potassium applied as potassium chloride (45%) at 60 kg ha^−1^ during the sowing period. Before sowing, winter wheat (Jintai 182 variety) was cultivated during the experimental study, and the sowing rate was 95 kg ha^−1^. Winter wheat was planted on September 31, 2017 and on October 1, 2018, while harvesting was done on June 15, 2018 and June 21, 2019, respectively. Data were collected from the field with an interval of 20–25 days during the months of March, April, and May. All other agricultural practices, such as weed control, irrigation, disease, and pesticide application, were accomplished timely on the basis of crop growth stage and demand according to the conventional practices adopted in Shanxi Province.

### Physiological traits of photosynthesis

As described by Ahmed et al. (2018), the photosynthetic rate (*P*_n_), stomatal conductance (*G*_s_), intercellular CO_2_ absorption (*C*_i_), and the transpiration rate (*T*_r_) of winter wheat under the nitrogen levels, nitrogen ratios, and N timings were examined using the Li-6400 convenient photosynthesis method (LI-COR Inc., Lincoln, NE, USA), prepared with a LED leaf chamber. To determine the photosynthesis traits in each treatment, two fully prolonged flag leaves per plot of winter wheat crop were selected at different stages, i.e., jointing, flowering, and grain filling. All the calculations were conducted from 10:00 to 11:10 a.m. on a clear sunny day under a CO_2_ concentration of 400 mol mol^−1^.

### Determination of chlorophyll content measured with SPAD 502

Soil–plant analysis development (SPAD) readings were taken with a Minolta SPAD-502 m (relative value from 0 to 100). The winter wheat leaf was dignified from the tip angle of the leaf to the leaf sheath, and the chlorophyll content was measured. The leaf was repeated three times. The average can be roughly measured as chlorophyll content.

### Spectrophotometric method

Winter wheat was harvested with two leaves instantly placed into a sealed bag, each processing collection of three, in time with measurements of photosynthesis. A weight of 0.08 g was precisely taken, added into a 25-ml volumetric flask with 80% acetone before sealing, and then left in the dark for 24 h. A Shimadzu UV-1800 UV–Vis spectrophotometer was used to measure the absorbance at 470, 663, 645, and 652 nm. The contents of four pigments, namely, chlorophyll A, chlorophyll B, total chlorophyll, and carotenoid, were calculated in accordance with Wang (2018).


(Eq. 1) $$\text{Ca }=\;13.95A_{665}\;-\;6.88A_{649}$$

(Eq. 2) $$\text{Cb }=\;24.96A_{649}\;-\;7.32_{665}$$

(Eq. 3) $$\text{Cx.c }=\;(1,000A_{470}\;-\;2.05\text{Ca}-114\text{Cb})/245$$

(Eq. 4) $$C_{t}\;=\text{ Ca}+\text{Cb}$$

Where Ca and Cb are the chlorophyll a (Chl a) and chlorophyll b (Chl b) contents, respectively; Cx.c is the carotenoid content, and *C*_t_ is the total chlorophyll content. *A*_665_, *A*_649_, and *A*_470_ denote the absorbance at 665, 649, and 470 nm, correspondingly.

### Statistical analysis

The data presented in this study were the mean of three replicates. All data were analyzed using ANOVA with randomized complete block design (RCBD). The significance of each source was determined with an *F*-test. Duncan’s multiple range test (DMRT) was conducted using a *post-hoc* mean separation test (*p*< 0.05). Comparisons between treatments were made on the basis of a significant change in the least significance difference (LSD; *p*< 0.05). The Shapiro–Wilk test was used to evaluate the normality of variance before carrying out the ANOVA. Microsoft Excel 365 was used for data calculation. All statistical analysis was completed using SPSS version 19.0 and SAS 9.3 (SAS Institute, Cary, NC, USA).

## Results and discussion

### Effect of nitrogen ratios and nitrogen timing on physiological traits

**Figure 2:**
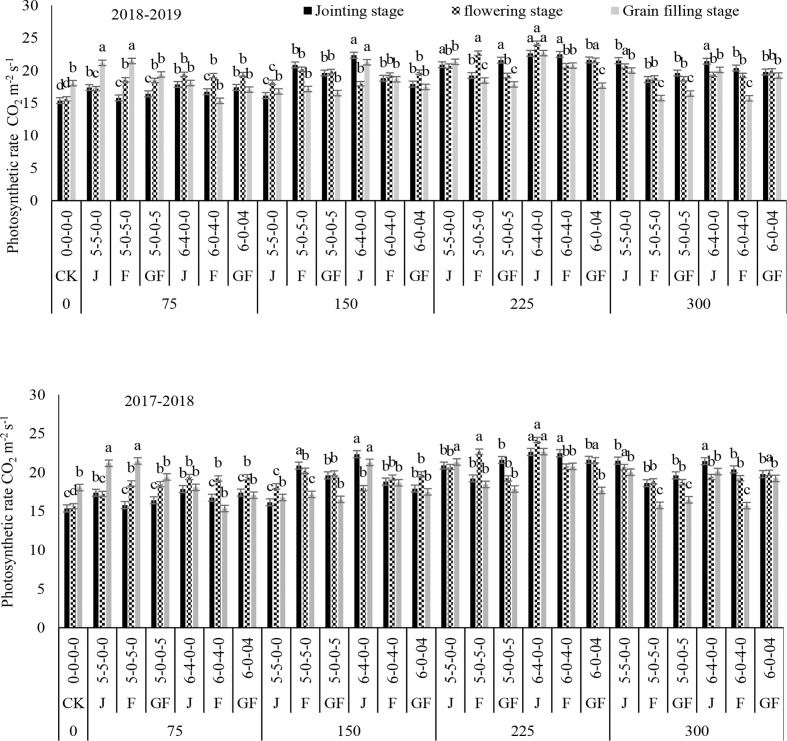
Effect of nitrogen fertilizer management on the photosynthetic rate of winter wheat. *JS*, jointing stage; *FS*, flowering stage; *GF*, grain filling stage. Different letters in above bars indicate a significant difference (p>0.05).

**Figure 3:**
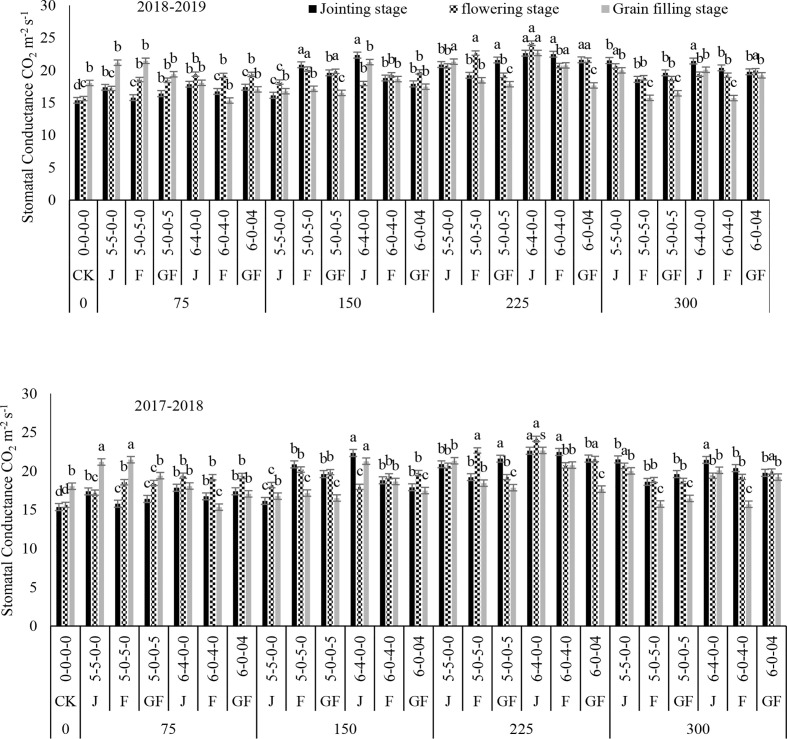
Effect of nitrogen fertilizer management on the stomatal conductance of winter wheat. *JS*, jointing stage; *FS*, flowering stage; *GF*, grain filling stage. Different letters in above bars indicate a significant difference (p>0.05).

**Figure 4:**
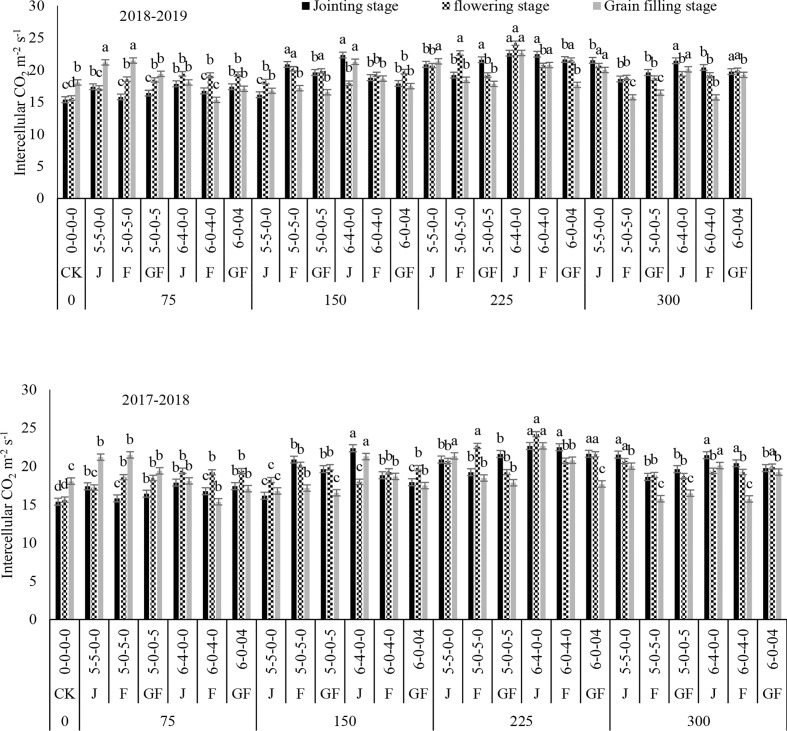
Effect of nitrogen fertilizer management on the intercellular CO_2_ concentration of winter wheat. *JS*, jointing stage; *FS*, flowering stage; *GF*, grain filling stage. Different letters in above bars indicate a significant difference (p>0.05).

**Figure 5:**
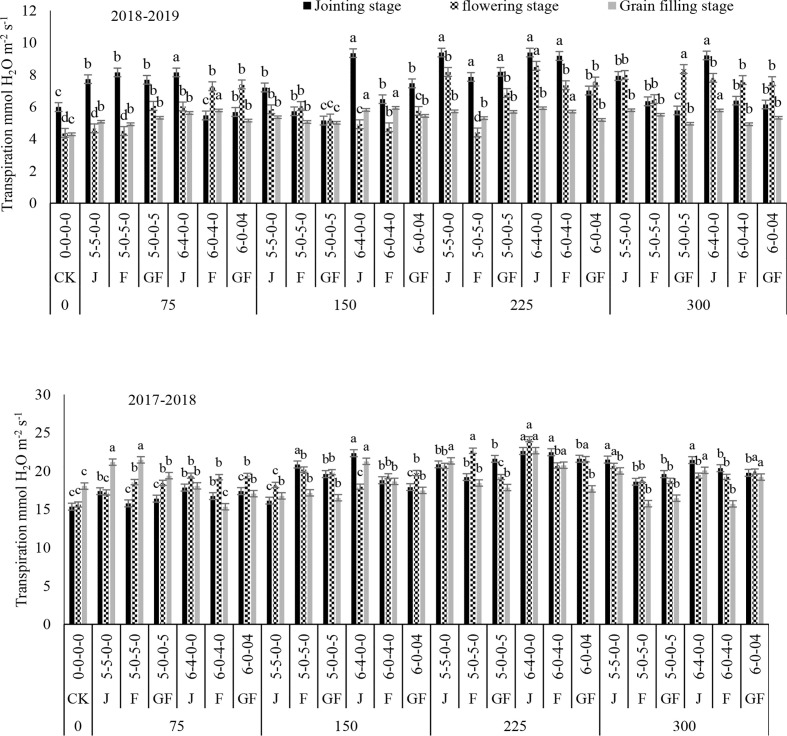
Effect of nitrogen fertilizer management on the transpiration rate of winter wheat. *JS*, jointing stage; *FS*, flowering stage; *GF*, grain filling stage. Different letters in above bars indicate a significant difference (p>0.05).

Nitrogen treatments significantly influenced the net photosynthetic rate (*P*_n_), stomatal conductance (*G*_s_), intercellular CO_2_ concentration (*C*_i_), and the transpiration rate (*T*_r_) in both study years (**Figures 2**–**5**). The maximum values for *P*_n_, *G*_s_, *C*_i_, and *T*_r_ during the different growth stages were recorded for 300 kg N ha^−1^, followed by 225 kg N ha^−1^. Higher values of these traits were noted during the flowering stage, followed by the jointing stage at the 6:4 ratio. However, 300 kg N ha^−1^ showed statistically the same or a lesser difference compared to 225 kg N ha^−1^. However, minimum values of *P*_n_, *G*_s_, *C*_i_, *T*_r_ were noted for 0 kg N ha^−1^, followed by 75, 150, and 225 kg N ha^−1^. In comparison to the control, 300 kg N ha^−1^ increased the *P*_n_ by 30% and 25.24%, *G*_s_ by 50% and 35%, and *C*_i_ by 28% and 23.50% in 2018 and 2019, respectively, during the flowering stage at the 6:4 ratio. On the other hand, the highest values of *T*_r_ observed were 8.69 and 9.39 mmol H_2_O m^−2^ s^−1^ at 225 kg N ha^−1^ during the jointing stage of winter wheat in 2018 and 2019, respectively, compared to the control. There was no significant variance between the nitrogen ratios of 6:4 under treatment with 225 kg N ha^−1^ and of 5:5 under treatment with 300 kg N ha^−1^. Analysis of variance showed that the N rates and ratios had a considerable impact on the photosynthetic traits (**Table 2**).

**Table 2:** Significance of the *F*-value from ANOVA of the various parameters of winter wheat affected by nitrogen management.

Parameter	N rates (*N*)	Ratios (*R*)	*N* × *R*
Photosynthetic rate	101.01***	2.95*	4.80***
Stomatal conductance	115.55***	30.74***	8.56***
Intercellular CO_2_	1,445.33***	39.82***	71.46***
Transpiration rate	23.92***	10.15***	3.05***
SPAD	7.73***	0.88 NS	1.87**
Chl a content	35.09***	1.10 NS	1.03 NS
Chl b content	21.62***	0.50 NS	0.63 NS
Total chlorophyll	71.05***	5.75***	2.10**
Carotenoids	18.65***	0.49 NS	0.29 NS

*, **, and *** represent significance levels at alpha 0.05, 0.01, and 0.001, respectively, obtained using honestly significant difference (HSD) test.

NS, non-significant; SPAD, soil–plant analysis development.

Increased values of *P*_n_, *G*_s_, *T*_r_, and *C*_i_ of the physiological traits of winter wheat were observed in our current study, which could be due to the optimal temperature and rainfall throughout its growing stages. The photosynthetic rate was affected by nitrogen fertilization and appropriate consumption of nitrogen fertilizer significantly increased the grain yield of the winter wheat crop (Du et al., 2019; Ziadi et al., 2010). Treatment with 225 kg N ha^−1^ revealed higher *P*_n_, *G*_s_, *T*_r_, and *C*_i_ values at the jointing, flowering, and filling stages when nitrogen was added at the jointing stage. The application time clearly affected the *P*_n_ or *G*_s_ of winter wheat, and a higher *P*_n_ rate was found at the growing stage when the application was done during the flowering stage and grain filling stages, which could be related to the higher transmittance at the early growing stages, assisting winter wheat crops with increasing their leaf area and accumulating additional sunshine. This might be due to the climate conditions and the higher chlorophyll contents (Weih et al., 2016), leaf area index (Wolf et al., 2003), and the net photosynthetic rate (Peltonen, 1992). In the present study, the values of *P*_n_, *T*_r_, *C*_i_, and *G*_s_ were enhanced with the increase of nitrogen, and maximum values were recorded with the 300- and 225-kg N ha^−1^ treatments. The results of this study suggested that nitrogen application at 225 kg ha^−1^ is the most favorable for the stomatal gas exchange and photosynthetic traits. Some published findings also confirmed that the photosynthetic rates of leaves increase with nitrogen enhancement until a threshold level is reached (Aranda et al., 2004; Long et al., 2006).

### Effect of nitrogen fertilizer on soil–plant analysis development

**Figure 6:**
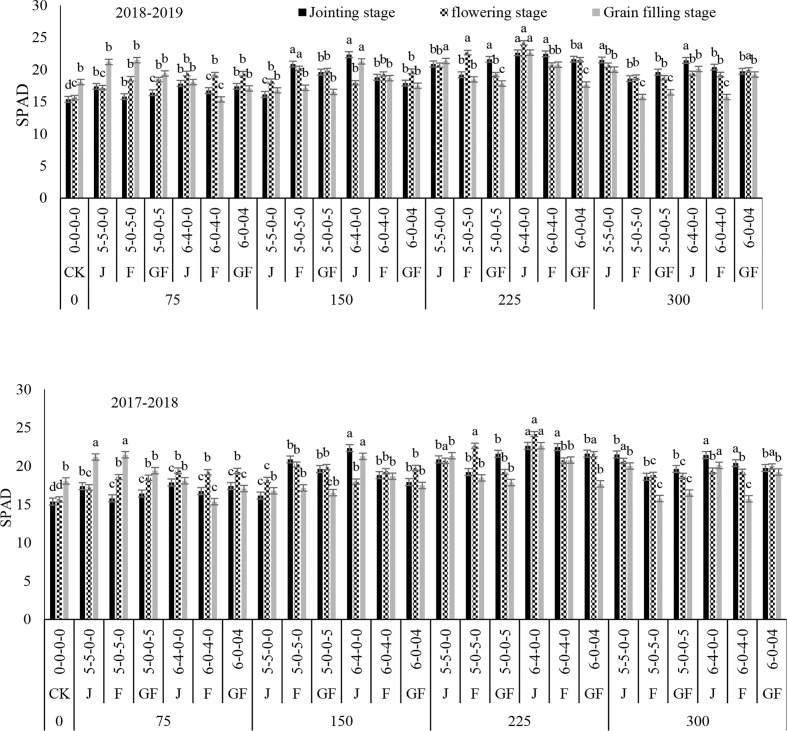
Effect of nitrogen fertilizer management on the SPAD 502 of winter wheat. *SPAD*, soil–plant analysis development; *JS*, jointing stage; *FS*, flowering stage; *GF*, grain filling stage. Different letters in above bars indicate a significant difference (p>0.05).

The SPAD value during the first year significantly increased and decreased in the second year with the increase of the nitrogen rates at the different growth stages (**Figure 6**). Maximum results were observed with treatment of 225 kg N ha^−1^ at the 6:4 ratio. The average increases were from 18.41% to 20.00% at the jointing stage, from 10.89% to 13.62% at the flowering stage, and from 11.48% to 10.06% at the grain filling stage. These results demonstrated that a high amount of nitrogen fertilizer will prime the decline of chlorophyll content in the flag leaf of winter wheat. The SPAD value was basically consistent with the chlorophyll content, which exhibited a tendency of an increase first and then a decline with the increase of the nitrogen rates. The differences among the treatments were measured at the<0.05 probability levels.

**Figure 7:**
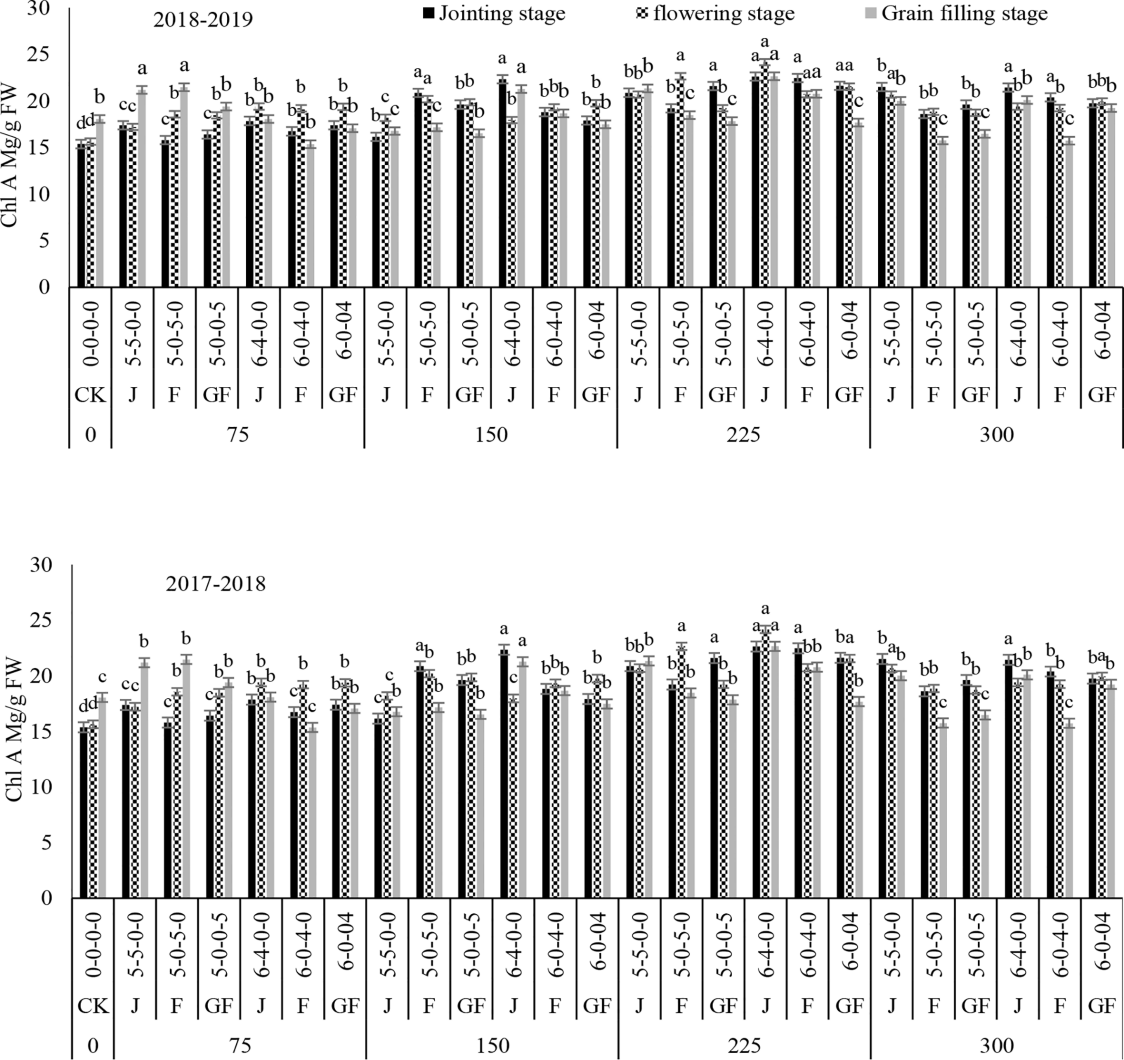
Effect of nitrogen fertilizer management on the Chl A content of winter wheat. *FW*, fresh weight; *JS*, jointing stage; *FS*, flowering stage; *GF*, grain filling stage. Different letters in above bars indicate a significant difference (p>0.05).

**Figure 8:**
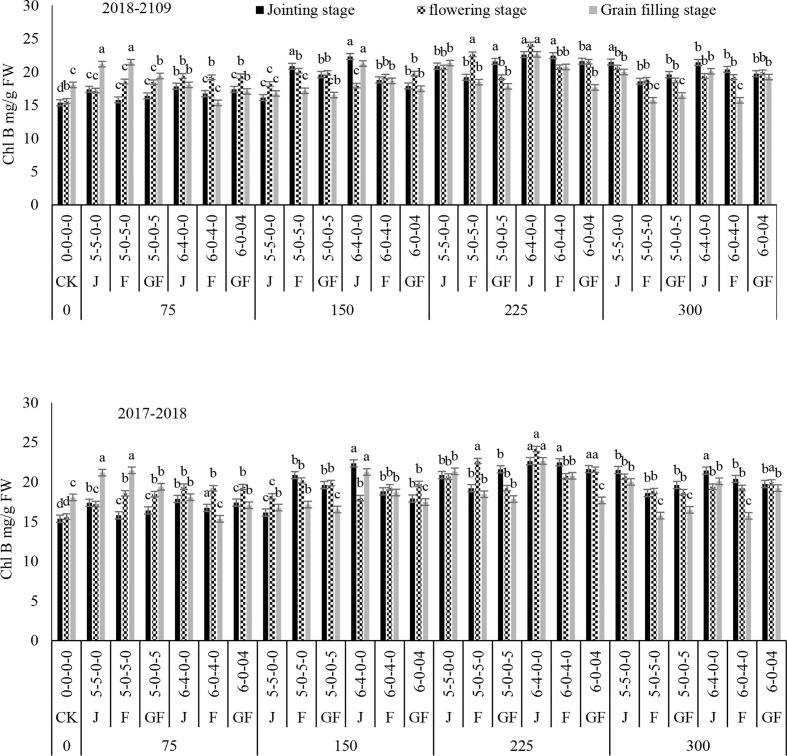
Effect of nitrogen fertilizer management on the Chl B content of winter wheat. *FW*, fresh weight; *JS*, jointing stage; *FS*, flowering stage; *GF*, grain filling stage. Different letters in above bars indicate a significant difference (p>0.05).

**Figure 9:**
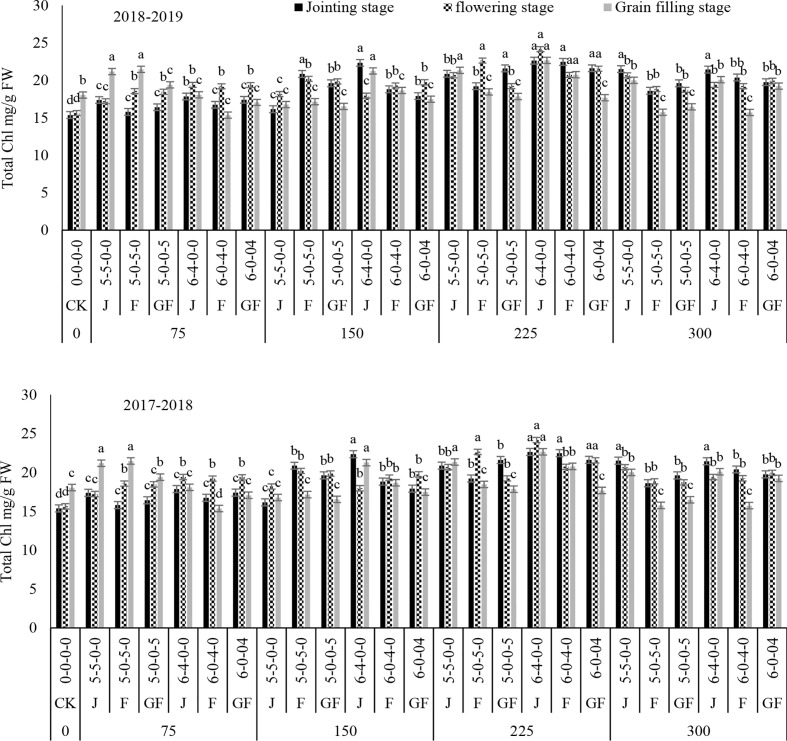
Effect of nitrogen fertilizer management on the total chlorophyll content of winter wheat. *FW*, fresh weight; *JS*, jointing stage; *FS*, flowering stage; *GF*, grain filling stage. Different letters in above bars indicate a significant difference (p>0.05).

**Figure 10:**
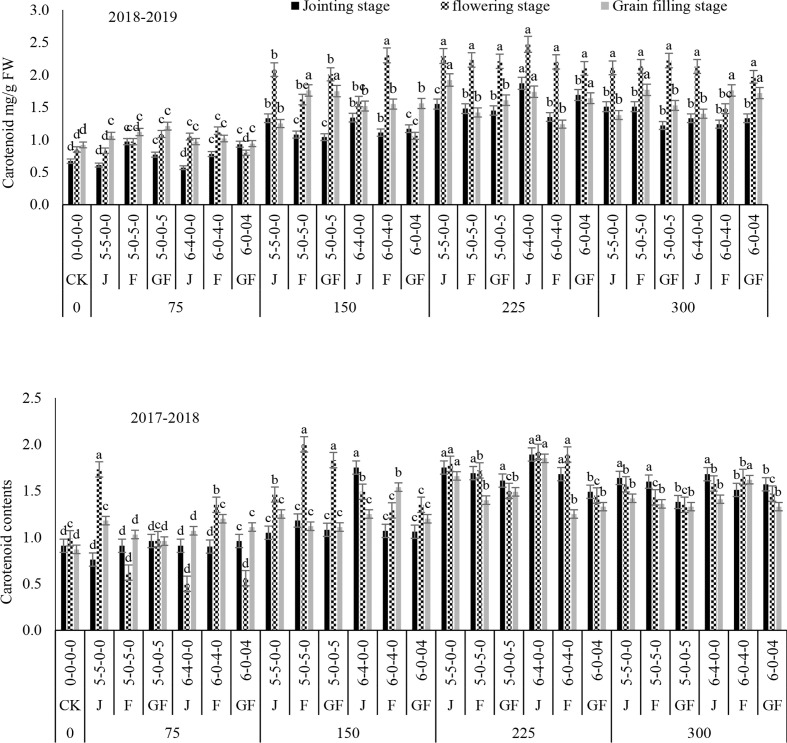
Effect of nitrogen fertilizer management on the carotenoid content of winter wheat. *JS*, jointing stage; *FS*, flowering stage; *GF*, grain filling stage. Different letters in above bars indicate a significant difference (p>0.05).

Numerous investigations have noted a strong link between SPAD and the N levels (Tahir and Nakata, 2005). For many plant species, the relationships between the total chlorophyll content and SPAD studies have been identified (Du et al., 2019; Mielke et al., 2012). Our results showed that pre-sowing and topdressing nitrogen application significantly augmented the SPAD. The SPAD value in treatment with 225 kg N ha^−1^ at a 6:4 ratio, when N was applied at the jointing stage, was significantly higher than that in treatment with 150 kg N ha^−1^ at a ratio of 5:5 when the fertilizer was applied at the flowering stage in both years as compared to 300 kg N ha^−1^ (**Figures 2**–**10**). Our finding is in line with those of studies in cereal crops by Wang (2018), Hawkesford et al. (2013), and Yingkui et al. (2016). At the initial growth periods from tillering to jointing, chlorophyll analyses usually indicated non-significant differences across the ecotypes and nitrogen application rates. Gao et al. informed that a grain yield response in winter wheat might be estimated for meter analyses of less than 42; the authors discovered that SPAD measurements did not differ at the jointing stage, prior to topdressing nitrogen application (Gao et al., 2018). In our investigation, the nitrogen applications increased dramatically in the various growth stages at the 6:4 ratio. When compared to control treatment CK, the winter wheat SPAD values with the 225-kg N ha^−1^ treatment and the 6:4 ratio were higher than those under the 5:5 ratio, which were 18.41%, 10.89%, and 11.48% and 20.00%, 13.62%, and 10.06%, respectively, in both years (**Figures 2**–**10**). The results matched those of a previous study by Kim et al., in which the leaf greenness of SPAD was significantly related to Chl a absorption in two rice varieties with diverse leaf colors. Therefore, fluctuations in leaf greenness are probably due to the absorption of chlorophyll. The variances in leaf greenness determined by the SPAD values have also been described in field crops (Weih et al., 2016; Papastylianou, 1984). The results from our study generally suggest that SPAD measurements at a crucial time are undoubtedly influenced by the plant nitrogen status and are related to the leaf area index, nitrogen content, and grain production in winter wheat plants. A recommendation for nitrogen application based on the SPAD results may be challenging; however, as key SPAD assessments may differ between years, the N timings, N ratios, and the soil characteristics and biochemical traits are affected by nitrogen fertilizer.

### Effect of nitrogen fertilizer on chlorophyll a, chlorophyll b, and total chlorophyll contents

The contents of Chl a and Chl b of winter wheat leaves were significantly greater with 300 and 225 kg N ha^−1^ in both years when compared to the control (**Figures 7**, **8**). In comparison to the control treatments, 225 kg N ha^−1^ increased the contents of Chl a and Chl b by 68.57% and 250%, respectively, at the jointing stage, followed by the flowering stage under the 6:4 ratio. Our outcomes designated that the Chl a process is superior to that of Chl b parallel to the control. The application of nitrogen fertilizer significantly improved Chl a, and the capability of the jointing stage under the growth process was higher than that of the flowering and filling stages because, at the jointing stage, plants were healthy and strong and chlorophyll was available in higher quality.

Compared with the control, fertilizer treatment at 225 kg ha^−1^ significantly increased the total chlorophyll (**Figure 9**). The overall trend in total chlorophyll was consistent between treatments and ratios. Initially, the total chlorophyll increased from the jointing stage and then declined over starting with the grain filling stage. Subsequently, the total chlorophyll contents increased with treatment of 225 kg N ha^−1^ at the ratio of 6:4, with overall increases of 85.89%–109.22%, 75.21%–80.78%, and 57.5–105.82% under 300, 150, and 75 kg N ha^−1^, respectively, in both years. However, ratios of 5:5 at the jointing stage under treatment with 300 kg N ha^−1^ and 6:4 at the jointing stage under treatment with 150 kg N ha^−1^ showed no significant difference in the total chlorophyll content. These results demonstrated that, with nitrogen application during different stages, a higher chlorophyll content can be preserved at the radical growing stages of winter wheat and that the flag leaf stage is the optimal stage for topdressing the fertilizer. Variance analysis showed that the nitrogen application rate and ratios had significant effects on the total chlorophyll content and that their interaction was also significant (**Table 2**).

Overall, the leaves in the experimental units differed significantly in color, ranging from shaded green to light brown and yellowish at the time of measurement. The results demonstrated that, in winter wheat plants on different ratios and N timings, the contents of Chl a, Chl b, total chlorophyll, and carotenoid first increased with the increase of the nitrogen rate and then decreased in both years as compared to those of CK (**Figures 2**–**10**). Chlorophyll was the main pigment in winter wheat plants and ranged from 5.82 to 6.96 mg/g fresh weight (FW), with Chl a ranging from 1.74 to 2.86 mg/g FW and Chl b from 7.56 to 9.75 mg/g FW. The Chl a-to-Chl b ratio was usually approximate to the various nitrogen treatments and ratios and the N timing. The *C*_t_ content was in the mean range of 1.74–2.47 mg/g FW under the ratio of 6:4 with treatment (225 kg N ha^−1^) from the jointing to the grain filling stage, when nitrogen was applied during the jointing stage in both years, as compared to CK. According to Mielke et al. (2012), Wang (2018), and Sicher and Bunce (2001), winter wheat plants have higher Chl a, Chl b, and carotenoid levels than CK during the jointing to the grain filling stage. Numerous investigations on various types of crops have described comparable outcomes (Gao et al., 2018; Baig et al., 2005). The numerous uses of nitrogen include promoting photosynthesis, enhancing tissue strength, and reducing plant transpiration rates.

Our results are in agreement with those of Gao et al. (2018), whose significance analysis confirmed that the chlorophyll content of winter wheat leaves significantly increased at the jointing and booting stages compared with that in the control (*p*< 0.05) (Gao et al., 2018; Tian et al., 2014; Yang et al., 2017). Skudra and Ruza (2017) has revealed that the leaf chlorophyll contents during jointing and flag leaf at diverse periods were increased by 3.3%–6.8% and 3.0%–23.4%, correspondingly, compared to those during the early flowering stage. When nitrogen was applied at the jointing stage, the Chl a and Chl b contents decreased slowly with the growing process by withholding nitrogen up to the flowering stage. Chlorophyll is essential in photon concentration, diffusion, and transportation and is closely associated with the *P*_n_ in leaves (Xing et al., 2018). Increasing the quantity of the nitrogen fertilizer can increase the chlorophyll content in plant leaves, elongating the period in which the photosynthetic rate is higher and consequently improving photosynthetic performance (Ahmed et al., 2018; Wang, 2018). In our study, pre-sowing and topdressing nitrogen applications significantly augmented the chlorophyll content of winter wheat plants. Under the jointing stage, the chlorophyll content was greater than that during the flowering and grain filling stages. Moreover, the 6:4 ratio with N timing during the jointing stage was higher than the 5:5 ratio under treatment with 225 kg N ha^−1^. This outcome demonstrated that topdressing nitrogen application to closely compete with the nitrogen supplies of both the N timing and treatments and ratios of winter wheat finally contributes to dry matter accumulation, subsequently to a greater N supply in the leaves, finally leading to significantly greater grain yields.

### Effect of nitrogen fertilizer on carotenoid content

The carotenoid contents of winter wheat in all nitrogen treatments and ratios were significantly greater than that of the control at the jointing stage. Higher results were obtained with the ratio of 6:4 at 225 kg N ha^−1^ in the jointing and flowering stages (**Figure 10**). The carotenoid contents following from the jointing to the grain filling stage at the different growth stages were increased by 78.88%–62.89% and 11.45%–53-94%, respectively, with 225 kg N ha^−1^, while nitrogen was suppressed until the grain filling stage. The ANOVA results showed that nitrogen treatment had a significant effect on the carotenoid content, while the N ratio and the interaction of the nitrogen level and ratio were not significant (**Table 2**). Carotenoids are essential pigments in photosynthesis and provide an indication of the leaf function and structure when several environmental factors are at work (Liu et al., 2019; Ahmed et al., 2018). Favorable climatic conditions and greater chlorophyll concentrations can possibly be of concern (Tian et al., 2014; Nikolic et al., 2002; Megda, 2009). Carotenoids are critical pigments of photosynthesis and are good indicators of leaf functions under the harmful influence of different environmental agents (Zhang et al., 2010); they comprise a crucial parameter for observing the uptake of nitrogen in winter wheat (Papastylianou, 1984). Some published findings also confirmed that leaf carotenoids increase with increasing nitrogen doses.

## Conclusions

Nitrogen application of 225 kg ha^−1^ at a ratio 6:4:0:0 significantly influenced the photosynthetic traits, leaf chlorophyll, and physiological characteristics of winter wheat. The splitting of the 225-kg ha^−1^ dose of nitrogen at a 6:4:0:0 ratio, 60% before sowing + 40% during the jointing stage, effectively improved the SPAD value, the Chl a, Chl b, total chlorophyll, and carotenoid contents, and the stomatal conductance (*G*_s_), photosynthetic rate (*P*_n_), intercellular CO_2_ (*C*_i_), and transpiration rate (*T*_r_) of winter wheat crops. The findings of the present study could be useful to developing the best nitrogen rate under appropriate N ratios for winter wheat production. The results support knowledge of nitrogen fertilizer management to maximize winter wheat productivity considering the conditions qualified in the current growing season. This study is the first attempt on the effects of applying different nitrogen ratios and timings during the growth stages on the physiological and biochemical traits of winter wheat in Shanxi, China. Additionally, the nitrogen doses and ratios could meritoriously increase the photosynthetic characteristics, ultimately improving the grain yield of winter wheat.

## Data availability statement

The original contributions presented in the study are included in the article/supplementary material. Further inquiries can be directed to the corresponding authors.

## Author contributions

MK, CW, MF, WY, YC, and HS contributed to the experimentation and other research-associated works. RN, KK, and KS reviewed and edited the manuscript. WY, MH, and WFAM contributed to the English proof reading. All authors contributed to the article and approved the submitted version.

## Funding

This work was funded by the National Natural Science Foundation of China (31871571, 31371572); the Scientific and Technological Innovation Fund of Shanxi Agricultural University (2018YJ17, 2020BQ32); the Higher education Project of Scientific and Technological Innovation in Shanxi (2020L0132); and the Key Technologies R&D Program of Shanxi Province (201903D211002). This project was also supported by the Outstanding Doctor Funding Award of Shanxi Province (SXYBKY2018040). Supported by the earmarked fund for Modern Agro-industry Technology Research System (2022-07).

## Conflict of interest

The authors declare that the research was conducted in the absence of any commercial or financial relationships that could be construed as a potential conflict of interest.

## Publisher’s note

All claims expressed in this article are solely those of the authors and do not necessarily represent those of their affiliated organizations, or those of the publisher, the editors and the reviewers. Any product that may be evaluated in this article, or claim that may be made by its manufacturer, is not guaranteed or endorsed by the publisher.

## References

[B1] Ahmed S. Raza M. Zhou T. (2018). Responses of soybean dry matter production, phosphorus accumulation, and seed yield to sowing time under relay intercropping with maize. Agronomy 8, 1–11. doi: 10.3390/agronomy8120282

[B2] Aranda I. Gil L. Pardos J. A. (2004). Improvement of growth conditions and gas exchange of Fagus sylvatica L. seedlings planted below a recently thinned Pinus sylvestris L. stand. Trees 18 (2), 211–220.

[B3] Baig M. J. Anand A. Mandal P. K. (2005). Irradiance influences contents of photosynthetic pigments and proteins in tropical grasses and legumes. Photosynthetica. 43, 47–53. doi: 10.1007/s11099-005-7053-5

[B4] Bertheloot J. Martre P. Andrieu B. (2008). Dynamics of light and nitrogen distribution during grain filling with in wheat canopy. Plant Physiol. 148, 1707–1720. doi: 10.1104/pp.108.124156 18799664 PMC2577274

[B5] Chandio A. A. Shah M. I. Sethi N. Mushtaq Z. (2022). Assessing the effect of climate change and financial development on agricultural production in ASEAN-4: the role of renewable energy, institutional quality, and human capital as moderators. Env. Scie Poll. Res. 29, 13211–13225. doi: 10.1007/s11356-021-16670-9 34585355

[B6] Chowdhury M. Kaium M. A. Hasan M. M. Bahadur (2021). Evaluation of drought tolerance of some wheat (Triticum aestivum l.) genotypes through phenology, growth, and physiological indices. J. Agro. 11, 1782–1792. doi: 10.3390/agronomy11091792

[B7] Du X. Xi M. Kong L. (2019). Split application of reduced nitrogen rate improves nitrogen uptake and use efficiency in sweet potato. Scient. Rep. 9, 1–11. doi: 10.1038/s41598-019-50532-2 PMC6773731 31575958

[B8] Fan M. Shen J. Yuan L. (2011). Improving crop productivity and resource use efficiency to ensure food security and environmental quality in china. J. Exp. Botany. 63, 13–24. doi: 10.1093/jxb/err248 21963614

[B9] FAO WHO (2009) "Principles and methods for the risk assessment of chemicals in food." Environmental Health Criteria, 240.

[B10] Gao M. Liu Y. Dong Y. (2018). Photosynthetic and antioxidant response of wheat to di (2-ethylhexyl) phthalate (DEHP) contamination in the soil. Chemosphere. 209, 258–267. doi: 10.1016/j.chemosphere.2018.06.090 29933162

[B11] Hirel B. Thierry T. Peter L. (2011). Improving nitrogen use efficiency in crops for sustainable agriculture. Sustain. 3, 1452–1485. doi: 10.3390/su3091452

[B12] Hawkesford MJ Araus JL Park R Calderini D Miralles D Shen T. (2013). Prospects of doubling global wheat yields. Food Energy Secur. 2 (1), 34–48.

[B13] Jialing Y. Wu J. (2018). The sustainability of agricultural development in china: the agriculture environment nexus. Sust. Bility. 10, 10: 1–10: 9. doi: 10.3390/su10061776

[B14] Liu Z. Gao F. Yang J. (2019). Photosynthetic characteristics and uptake and translocation of nitrogen in peanut in a wheat-peanut rotation system under different fertilizer management regimes. Front. Plant Sci. 10, 1–11. doi: 10.3389/fpls.2019.00086 30792727 PMC6374608

[B15] Li W. Xue C. Pan X. (2018). Application of controlled-release urea enhances grain yield and nitrogen use efficiency in irrigated rice in the yangtze river basin China. Front. Plant Sci. 9, 13–25. doi: 10.3389/fpls.2018.00999 30073007 PMC6060282

[B16] Long S. P. Zhu X. G. Naidu S. L. Ort D. R. (2006). Can improvement in photosynthesis increase crop yields? Plant Cell Environ. 29 (3), 315–330. doi: 10.1111/j.1365-3040.2005.01493.x 17080588

[B17] Mahjourimajd S. Kuchel S. Langridge F. (2016). Evaluation of australian wheat genotypes for response to variable nitrogen application. J. Plant Soil 399, 247–255. doi: 10.1007/s11104-015-2694-z

[B18] Man J. Shi Y. Yu Z. (2015). Dry matter production, photosynthesis of flag leaves and water use in winter wheat are affected by supplemental irrigation in the huang-huai-hai plain of China. PLo. One 10, e0137274. doi: 10.1371/journal.pone.0137274 PMC4559388 26335019

[B19] Megda M. M. (2009). Response of wheat cultivars to nitrogen in relation to the sources and times of application under planting sprinkler irrigation. Sci. Agro. Techno. 33, 1055–1060.

[B20] Mielke M. S. Schaffer B. Schilling A. C. (2012). Evaluation of reflectance spectroscopy indices for estimation of chlorophyll content in leaves of a tropical tree species. Photosynthetica. 50, 343–352. doi: 10.1007/s11099-012-0038-2

[B21] Mingnanet Q. Chu X. Xin Z. (2017). Leaf photosynthetic parameters related to biomass accumulation in a global rice diversity survey. Plan. Physio. 175, 248–258. doi: 10.1104/pp.17.00332 PMC5580745 28739819

[B22] Nikolic O. Zivanovic T. Jelic M. (2002). Interrelationships between grain nitrogen content and other indicators of nitrogen accumulation and utilization efficiency in wheat plants. J. Agr. Res. 72, 11–116.

[B23] Oldfield E. E. Bradford M. A. (2019). Global meta-analysis of the relationship between soil organic matter and crop yields. Soil. 5, 15–32. doi: 10.5194/soil-5-15-2019

[B24] Panhwar Q. A. Ali A. Naher U. A. Memon M. Y. (2019). Fertilizer management strategies for enhancing nutrient use efficiency and sustainable wheat production. In Org. far. 11, 17–39. doi: 10.1016/B978-0-12-813272-2.00002-1

[B25] Papastylianou I. (1984). Diagnosis of the nitrogen status of wheat at tillering and prognosis for maximal grain yield. Commun. Soil. Sci. Plant Anal. 15, 1423–1436. doi: 10.1080/00103628409367570

[B26] Peltonen J. (1992). Tissue nitrogen as a base for recommendations of additional nitrogen to spring wheat in southern Finland. Acta Agric. Scand. 42, 164–169. doi: 10.1080/09064719209417972

[B27] Sabagh A. Islam M. S. Skalicky M. Ali Raza M. Singh K. (2021). Salinity stress in wheat (Triticum aestivum l.) in the changing climate: Adaptation and management strategies. J. Front. Agr. 3, 661932. doi: 10.3389/fagro.2021.661932

[B28] Sahar Y. Allahyar F. Jahanfar D. (2012). Effect of split application of nitrogen fertilizer on growth and yield of hybrid rice. Advan. Environ. Biol. 6(9), 2485–2589.

[B29] Shah F. Wei W. (2019). Soil and crop management strategies to ensure higher crop productivity within sustainable environments. Sustainability 11, 1474–1485. doi: 10.3390/su11051485

[B30] Shi Y. Yu Z. (2008). Effects of nitrogen fertilizer rates and ratios of base and topdressing on wheat yield, soil nitrate content and nitrogen balance. Front. Agric. China 2, 181–189. doi: 10.1007/s11703-008-0027-1

[B31] Sicher R. C. Bunce J. A. (2001). Adjustments of net photosynthesis in solanum tuberosum in response to reciprocal changes in ambient and elevated growth CO_2_ partial pressures. Physiol. Plantarum. 112, 55–61. doi: 10.1034/j.1399-3054.2001.1120108.x 11319015

[B32] Skudra I. Ruza A. (2017). Effect of nitrogen and sulphur fertilization on chlorophyll content in winter wheat. Rural. Sust. Res. 37, 29–37. doi: 10.1515/plua-2017-0004

[B33] Svetla K. Nevenka G. Marina M. (2016). Uptake and utilization efficiency of nitrogen and phosphorus in barley genotypes. J. Cent. Euro. Agr. 17, 346–355. doi: 10.5513/JCEA01/17.2.1714

[B34] Tahir I. S. Nakata N. (2005). Remobilization of nitrogen and carbohydrate from stems of bread wheat in response to heat stress during grain filling. J. Agro. Scie 191, 106–115. doi: 10.1111/j.1439-037X.2004.00127.x

[B35] Tian Y. Zheng C. Chen J. (2014). Climatic warming increases winter wheat yield but reduces grain nitrogen concentration in East China. Plo. One 9, e95108. doi: 10.1371/journal.pone.0095108 PMC3988157 24736557

[B36] Wang L. (2018). Leaf photosynthetic function duration during yield formation of large-spike wheat in rainfed cropping systems. PeerJ. 6, e5532. doi: 10.7717/peerj.5532 30280014 PMC6166621

[B37] Wang D. (2018). Low recovery efficiency of basal fertilizer n in plants does not indicate high basal fertilizer n loss from split applied n in transplanted rice. Field. Crops. Res. 229, 8–16. doi: 10.1016/j.fcr.2018.09.008

[B38] Weih M. Pourazari F. Vico. G. (2016). Nutrient stoichiometry in winter wheat: Element concentration pattern reflects developmental stage and weather. Scienti. Rept. 6, 1–9. doi: 10.1038/srep35958 PMC5075900 27775050

[B39] Wolf N. Wolf A. Hoskins B. (2003). Dry matter analysis method. a report to the manure analysis. Cop. Ext. Publ. 3769, 1–2.

[B40] Xing X. Yuhan T. Mengran W. (2018). Effect of paclobutrazol application on plant photosynthetic performance and leaf greenness of herbaceous peony. Horticulturae. 4, 3–12. doi: 10.3390/horticulturae4010005

[B41] Xu H. C. Dai X. L. Chu J. P. (2018). Integrated management strategy for improving the grain yield and nitrogen-use efficiency of winter wheat. J. Integ. Agr. 17, 315–327. doi: 10.1016/S2095-3119(17)61805-7

[B42] Yang J. Zhou Q. Zhang J. (2017). Moderate wetting and drying increases rice yield and reduces water use, grain arsenic level, and methane emission. J. Crop Sci. 5, 151–158. doi: 10.1016/j.cj.2016.06.002

[B43] Yingkui Y. Yasuyuki I. (2016). Year-round forage yield stability through a system combining triple-maize crops with winter barley in kyushu, japan. Ameri. J. Agric. Biol. Scie. 11, 19–28. doi: 10.3844/ajabssp.2016.19.28

[B44] Zhang J. H. Jian L. U. Zhang J. B. (2010). Effects of nitrogen application rates on translocation of dry matter and nitrogen utilization in rice and wheat. Acta Agro. Sinica. 36, 1736–1742. doi: 10.1016/S1875-2780(09)60079-1

[B45] Ziadi N. Belanger G. Claessens A. (2010). Plant based diagnostic tools for evaluating wheat nitrogen status. Crop Sci. 50, 25–80. doi: 10.2135/cropsci2010.01.0032

[B46] Zuliang S. Fei W. Xiang L. (2018). Dry matter and nitrogen accumulation as affected by nitrogen fertilization and seeding rate in winter wheat. J. Agr. Frstry. 6, 50–59. doi: 10.11648/j.ajaf.20180603.13

