# Erratum: Nitrogen fertilizer application rates and ratios promote the biochemical and physiological attributes of winter wheat

**DOI:** 10.3389/fpls.2022.1123148

**Published:** 2023-01-10

**Authors:** Frontiers Production Office

**Affiliations:** Frontiers Media SA, Lausanne, Switzerland

**Keywords:** winter wheat, nitrogen fertilizer, photosynthetic traits, chlorophyll content, (SPAD)

Due to a production error, an author’s name was incorrectly spelled as “Hasan Mohamed”. The correct spelling is “Mohamed E. Hasan”. The publisher apologizes for this mistake.

The original version of this article has been updated.

